# Chronic cutaneous candidiasis in children: should we stop there? Report of two cases associated with auto-immune polyendocrinopathy syndrome type I

**DOI:** 10.1186/s12887-020-02030-y

**Published:** 2020-03-18

**Authors:** Basilice Mireille Minka, Aurélie Sibetcheu T, Suzanne Ngo Um Sap, Maryse Césarine Bissa

**Affiliations:** 1 Department of Pediatrics, Faculty of Medicine and Biomedical Sciences, University of Yaounde I, Yaounde, Cameroon; 2 Mother and Child Centre, Chantal Biya’s Foundation, Yaounde, Cameroon

**Keywords:** Polyendocrine syndrome, Auto-immune, Children, Candidiasis, Cameroon

## Abstract

**Background:**

Auto-immune polyendocrinopathy syndrome type I is a rare genetic disease, usually revealed by chronic superficial candidiasis and autoimmune endocrine dysfunction in childhood.

**Cases presentation:**

We report the cases of 2 children, a 4 years-11 months old boy and 13 years old adolescent, admitted and followed up in the endocrinology unit of the Mother and Child Centre of Chantal Biya’s Foundation for auto-immune polyendocrine syndrome type 1.

**Conclusion:**

The occurrence of chronic cutaneous candidiasis in a child should always imply endocrine screening, to exclude auto-immune polyendocrine syndrome type I.

## Background

Auto-immune Polyendocrine Syndromes (APS) are rare diseases characterized by the association of at least 2 endocrine deficiencies related to an auto-immune mechanism, and/or other non-endocrine diseases. Various major and minor pathologies are included in these syndromes, and determined its classification. The multiple clinical presentation and unpredictable evolution of this condition are sources of diagnostic challenges, especially in resource-limited setting.

## Cases presentation

### Case 1

A 4-years-11 months old boy presented to our consultation for a 5-day history of fatigue and a 3-day-history of fever. Since 5 months of age, he was presenting chronic cutaneous candidiasis, persistent despite administration of antifungals; he also had a 3-month history of dysphagia, bilateral and symmetrical inflammatory joints pains without joint deformation but progressive functional impotence treated by bed rest, massages and oral antalgics.

At the emergency unit, the patient was in pain and unable to walk. He had a hyperpigmented, disseminated non pruriginous macular rash, subcutaneous tender nodules on both limbs and an aphthous gingivolabial lesion. The diagnosis of acute rheumatic fever had been ruled out by laboratory tests, normal echocardiography, and no improvement on trial treatment. 10 days later, the case was reviewed by the pediatric endocrinologist, who diagnosed adrenal insufficiency, confirmed by 8 am cortisol levels of 15 ng/mL. Unfortunately, auto-antibodies levels could not be assessed due to financial constraint. After initiation of hydrocortisone, asthenia subsided and melanodermia reduced markedly. One month later, the patient developed global hypotonia and subcutaneous nodules reappeared in greater number (Gottron’s signs), as well as joints pains. These signs, in addition to the symmetrical and proximal muscle weakness of the pelvic girdle, bilateral and symmetrical inflammatory joints pains, dysphagia and elevation of serum levels of muscle-associated enzymes (LDH: 926 UI/L, CPK: 900 UI/L), were typical of dermatopolymyositis, our associated diagnosis. 2 months later, a multidisciplinary staff was held and the association of chronic mucocutaneous candidiasis, adrenal insufficiency and dermatopolymyositis was in favor of APS type 1. Identification of mutations of AIRE (Auto-immune Regulator) gene could not be done due to limited finances. 6 months later (the patient was lost to follow up), the patient was readmitted with severe sepsis and died in the 1st day of admission.

### Case 2

An adolescent of 13 years old was referred by a dermatologist for endocrinology review for diffuse and repeated cutaneous candidiasis since 5 months of age. The initial evaluation also revealed melanodermia, delayed puberty and stunting. Turner syndrome was our first diagnosis with panhypopituitarism as differential diagnosis. Pelvic ultrasound was normal. Hormonal dosages revealed central hypothyroidism (TSH 10UI/ml), Growth Hormone deficiency (low GH levels before and after stimulation), and adrenal insufficiency (8 am cortisol levels 10.7 ng/mL). Gonadotrophins Releasing Hormone, and gonadotrophins levels were normal. The bone age (8 years) was less than the chronological age. These results oriented us towards the diagnosis of APS type 1 (chronic candidiasis and Addison’s disease). Hormonal replacement therapy with hydrocortisone, levothyroxine and growth hormone was initiated with transient improvement. 1 month later, she was brought back for fatigue, severe headaches and signs of raised intracranial pressure. With the exception of diffuse cerebral atrophy, cerebral CT-scan was normal. Cerebrospinal fluid analysis identified *Cryptococcus neoformans*. She died several days later.

## Discussion and conclusion

APS are diseases characterized by the combination of at least 2 endocrine deficiencies related to an auto-immune mechanism, and sometimes a non-endocrine auto-immune disease [1]. It was previously classified by Neufeld and Blizzard into 4 types [2]. Currently, APS include 2 entities: APS type 1 and APS type 2 (type 2 englobes types 2, 3 and 4 of the Neufeld and Blizzard classification). Few cases have been reported in Africa; to our knowledge, it is the 2nd description of APS type 1 in children [3].

APS type 1 or APECED (Autoimmune Polyendocrinopathy Candidiasis Ectodermal Dystrophy), is an autosomal recessive disease, caused by composite homozygous or heterozygous mutations of AIRE (Auto-immune Regulator) gene. This gene located on 21q22.3 chromosome, encodes for a transcription factor controlling negative selection of autoreactive lymphocytes [4]. APS type 1 has been demonstrated to occur more frequently in patients with high consanguinity (Sardinia, Finland, Iranian Jewish) with estimated prevalence of 1/14400, 1/25000 and 1/9000 inhabitants respectively [5–7].. The symptoms usually starts during the 1st year of life and are fully developed before 20 years [8]. In the natural history, mucocutaneous candidiasis occurs before 5 years, followed by hypocalcemia related to hypoparathyroidism by 10 years of age, and patients develop Addison’s disease before 15 years [8, 9]. Diagnosis of APS type I requires 2 of the 3 major criteria of Whitaker triad (chronic mucocutaneous candidiasis, hypoparathyroidism, Addison’s disease).

As reported by many authors [3, 4, 9], mucocutaneous candidiasis was the inaugural disease in our patients. The occurrence of cryptococcal meningitis in the 2nd patient was unexpected. In fact, the absence of AIRE gene is responsible of the alteration of monocytes communication proteins, affecting the innate immune response of T-helper lymphocytes towards *Candida albicans* [10]. Furthermore, anti-interleukin 17 and 22 auto-immunization could contribute to fungal infections by altering antifungal defenses. Meanwhile, humoral immunity is intact, preventing the occurrence of systemic candidiasis [11].

Others pathologies have been associated to APS type I [12, 13]. Auto-immune hypophysitis, identified in our 2nd patient, is reported in 3% of cases of APS type I [1]. Dermatopolymyositis in a context of APS type I has never been described in the literature, to the best of our knowledge.

Long term survival of APS type 1 patients requires regular follow up and early detection of new endocrinopathies or auto-immune diseases. In our cases, diagnostic wandering, limited financial and technical means for exhaustive hormonal testing and adequate therapy led to a bad outcome.

Every chronic mucocutaneous candidiasis resistant to adequate antifungal treatment in a child should implicate further investigations for underlying endocrinopathies and other auto-immune diseases, indicative of APS type 1. These cases highlight the importance of a multidisciplinary approach in the diagnosis of patients with unusual clinical presentations. Despite poverty and the unavailability of some diagnostic tools, this rare pathology was identified, even if the outcomes was bad.

## Data Availability

Data sharing is not applicable to this article as no datasets were generated or analysed during the current study.

## Abbreviations

AIRE: Auto-immune Regulator
APECED: Auto-immune Polyendocrinopathy Candidiasis Ectodermal Dystrophy
APS: Auto-immune Polyendocrine Syndrome
GH: Growth Hormone
TSH: Thyroid Stimulating Hormone
